# Preference of spectral features in auditory processing for advertisement calls in the music frogs

**DOI:** 10.1186/s12983-019-0314-0

**Published:** 2019-05-10

**Authors:** Yanzhu Fan, Xizi Yue, Jing Yang, Jiangyan Shen, Di Shen, Yezhong Tang, Guangzhan Fang

**Affiliations:** 1 Department of Herpetology, Chengdu Institute of Biology, Chinese Academy of Sciences, No.9 Section 4, Renmin Nan Road, Chengdu, Sichuan 610041 People’s Republic of China; 2 University of Chinese Academy of Sciences, 19A Yuquan Road, Beijing, People’s Republic of China

**Keywords:** Auditory processing, Advertisement call, Event related potential (ERP), Spectral characteristic, Temporal characteristic, Frog

## Abstract

**Background:**

Animal vocal signals encode very important information for communication during which the importance of temporal and spectral characteristics of vocalizations is always asymmetrical and species-specific. However, it is still unknown how auditory system represents this asymmetrical and species-specific patterns. In this study, auditory event related potential (ERP) changes were evaluated in the Emei music frog (*Babina daunchina*) to assess the differences in eliciting neural responses of both temporal and spectral features for the telencephalon, diencephalon and mesencephalon respectively. To do this, an acoustic playback experiment using an oddball paradigm design was conducted, in which an original advertisement call (OC), its spectral feature preserved version (SC) and temporal feature preserved version (TC) were used as deviant stimuli with synthesized white noise as standard stimulus.

**Results:**

The present results show that 1) compared with TC, more similar ERP components were evoked by OC and SC; and 2) the P3a amplitudes in the forebrain evoked by OC were significantly higher in males than in females.

**Conclusions:**

Together, the results provide evidence for suggesting neural processing for conspecific vocalization may prefer to the spectral features in the music frog, prompting speculation that the spectral features may play more important roles in auditory object perception or vocal communication in this species. In addition, the neural processing for auditory perception is sexually dimorphic.

## Background

Vocal communication plays a crucial role in the survival and reproduction success in vocal animals such as birds, insects and anurans. In general, animal vocal signals encode diverse information about species, sexual receptivity, location, size and individual identity [1–3]. In the time domain, a natural vocalization typically contains a number of discrete components, appropriately ordered in time, each having specific spectral and temporal characteristics [4]. Accordingly, animal vocalizations provide a rich source of information which receivers must decode for species discrimination and individual recognition [5]. Previous studies show that the relationship between vocal signals and auditory processing is often consistent with the matched filter hypothesis [6], which holds that coevolution of signals and sensory systems should result in a good match between signal structure and the tuning of relevant sensory systems. For example, in zebra finches (*Taeniopygia guttata*), syllable diversity and male performance parameters such as spectral and temporal consistency rather than long song duration or high (directed) song rates are better predictors of which songs a female will find attractive [7].

The vocalization is both species-specific and individually distinct, and it functions in both territory defense and mate attraction [8]. For vocal animals, biotic noise sources from conspecific and heterospecific individuals are usually the major acoustic interference in many habitats [9, 10]. It is conceivable that, to reduce mutual masking, the signals of different species may be shifted by selection pressure to different frequency bands or spectral characteristics, so that species eventually avoid spectral overlap and hence occupy distinct acoustic niches [11]. Compared with other songbirds, the vocal repertoire of zebra finches includes more harmonic complexes with over 15 frequency components, and that differences in frequency separation and relative amplitude of each component lead to differences in pitch and timbre between individuals [12]. Similarly, the advertisement calls in some anuran species possess various spectral features different from each other among conspecific individuals so that these properties contribute toward individual recognition [13–15]. Thus, the spectral attributes of sounds might play important roles in vocal communication. At the neural level, different frequency components can be represented by activity in different frequency-tuned neural subpopulations or channels, i.e. tonotopic representation of sound [16]. Furthermore, vocalizations usually vary in temporal structure and these temporal properties can also play important roles in vocal communication [17]. Correspondingly, another fundamental aspect of auditory processing is neural synchrony to the temporal structure of sound such as envelope following [18] and frequency following [19] found in the instantaneous firing rate of auditory neurons. Interestingly, frequency resolution and temporal resolution for acoustic signals are inversely related to one another, both at the species and individual level in songbirds [20], implying the spectral and temporal features may contribute differently in vocal communication or perception of auditory object, i.e. the fundamental perceptual unit in hearing [21, 22]. Yet, there is still much that remains unknown about how auditory system represents the differences between these two features.

In anurans, survival and reproductive behaviors depend primarily on a listener’s ability to parse acoustic signals that convey species identity and individual information [23]. Usually, males are highly vocal and generally produce species-specific advertisement calls to attract females for breeding, as well as to deter rivals [24–26]. For species discrimination, either temporal information [5, 27] or spectral one [14, 28] may be more important in many anuran species. For individual recognition, the fundamental frequency and correlated spectral properties in advertisement calls of some species are often the most individually distinct call properties and contribute toward assigning calls to correct individuals [13–15, 29–31]. In contrast, female choices in some species are often mediated by temporal characteristics of calls [5, 32–34]. Interestingly, the temporal and spectral acoustic cues are used for sexual identity recognition and conveying female attractiveness respectively in *Xenopus laevis* [35]. These results suggest that the significance of temporal and spectral features of vocalizations is asymmetrical and species-specific for vocal communication. Numerous studies suggest that anurans have neural specializations for analyzing the temporal and spectral structures. In addition, anurans typically exhibit a small vocal repertoire and communicate in well-defined behavioral contexts making these species well suited for studies of auditory perception [36, 37]. However, it is still unknown how auditory system represents this asymmetrical and species-specific differences in temporal and spectral features of vocalizations observed in behaviors.

The Emei music frog (*Babina daunchina*) is a typical seasonal reproductive species in which males produce advertisement calls either from inside underground nest burrows or from outside burrows in the breeding season [38–41]. The resonant properties of the nest burrows modify call acoustics, such as extending note duration and decreasing note fundamental frequency, yielding two types of advertisement calls. Calls produced from inside the nests are highly sexually attractive (HSA) to females while those produced from open fields are of low sexual attractiveness (LSA) [40]. Females prefer HSA calls to LSA calls in phonotaxis experiments and males more likely to compete against HSA calls compared to LSA calls [40, 41], consistent with the idea that selective attention may be involved in anuran auditory perception [42, 43] and males can maximize fitness by adjusting competitive strategies to match female preferences and avoid the interference of other males [44]. These results also indicate differences in the temporal or spectral features of advertisement calls are easily recognized by the music frogs, providing an excellent model system for studying the neural mechanisms underlying auditory object perception of acoustic differences in vocalization. Moreover, compared with the temporal features, spectral properties may provide more sufficient information for individual recognition in this species [38], suggesting the spectral features may play important roles in vocal communication. Electrophysiological studies have shown that HSA and LSA calls can elicit significantly different event-related potential (ERP) components [45–48], suggesting ERP components can depict the differences in neural responses to temporal and spectral features of vocalization. In addition, the music frogs preferentially use the right ear to detect conspecific calls which conveys auditory information most strongly to the left auditory midbrain [49, 50], consistent with the idea that discrete brain structures are specialized for different functions [51]. Accordingly, it is logical to hypothesize that specific brain structures will be involved in auditory neural processing in this species.

ERP is the measured brain response to a specific sensory, cognitive or motor event [52], whose amplitudes and latencies can be used to examine processing efficiency and time course of information processing in the brain. Auditory ERPs generally consist of three main components (N1, P2 and P3) which peak at latencies of ~ 80 ms, ~ 200 ms and ~ 300 ms, respectively [53–57]. Functionally, N1 with negative peak is sensitive to selective attention [53]; P2 with positive peak is sensitive to the stimulus complexity and the subject’s familiarity with the sound [54]; while P3 can be divided into two general types: P3a elicited by novel deviant stimulus with passive paradigm and P3b (the conventional P3) elicited by the target stimulus with active paradigm [58]. P3a, also known as “novelty P300” [59], is a reflection of automatic detection of a different stimulus or stimulus relative novelty, i.e. novel or more salient differences between standard and deviant stimuli produce larger P3a waves [60]. In addition, familiar sounds evoke smaller P3a compared with unfamiliar ones [61]. Moreover, humanlike auditory ERP components, found in various taxa including non-human primates [62], mammals [63, 64] and anurans [45, 48, 65], may indicate similar brain functions because important neuroanatomical features have been conserved during vertebrate brain evolution [66, 67]. Since discrete brain regions may be specialized for different functions [51], the present study measured the amplitude and latency of each ERP component for the left and right hemispheres in response to three acoustic stimuli (the original advertisement call, OC; and its transformation version with temporal and spectral features preserved respectively, TC and SC) in order to investigate how auditory central nervous system represents the differences of these two call features in auditory neural processing. Furthermore, the fundamental perceptual unit in hearing is auditory object [21, 22], and that its neural representation must be based on information conveyed by one or more senses. Under these conditions we predicted that (1) more similar ERP components would be evoked by OC and TC if auditory processing of conspecific vocalization prefers to temporal features in the music frog; (2) alternatively, more similar ERP components would be evoked by OC and SC if the neural processing depends on spectral features primarily; and (3) ERP components will vary across brain structures such as various portions of a brain region.

## Materials and methods

### Animals and surgery

Sixteen adult frogs (8 males and 8 females) were captured from the Emei mountain area of Sichuan, China for the present experiments. Animal husbandry and laboratory animal care were the same as used in previous work and have been described elsewhere [49, 68, 69]. Briefly, the male and female frogs were separated by sex and were breeding in different plastic tanks (45 × 35 cm^2^ and 30 cm deep) which were paved with mud and water and the subjects were fed fresh live crickets every 3 days. The tanks were placed in a constant temperature room (23 ± 1 °C) that was maintained on a 12:12 light-dark cycle (lights on at 08:00). At the time of surgery, the mean mass and length of the subjects were 11.0 ± 0.6 g and 4.6 ± 0.1 cm respectively.

**Fig. 1:**
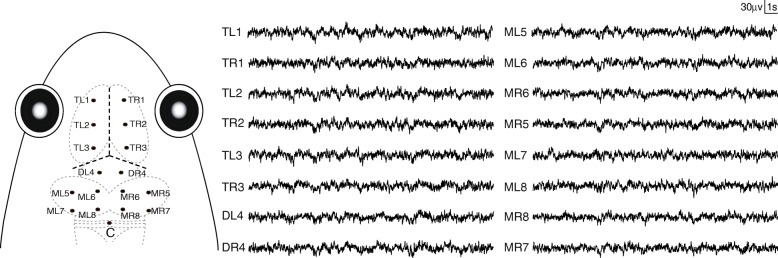
Electrode placements and their 20 s of typical EEG tracings. The intersection of the three dashed lines in bold in the frog head denotes the intersection of suture lines corresponding to lambda. The electrodes coordinates: TL1 (− 1.5, 3.8), TR1 (1.5, 3.8), TL2 (− 1.5, 2.4), TR2 (1.5, 2.4), TL3 (− 1.5, 1), TR3 (1.5, 1); DL4 (− 0.8, − 0.2), DR4 (0.8, − 0.2); ML5 (− 2.2, − 1.6), ML6 (− 0.8, − 1.6), MR6 (0.8, − 1.6), MR5 (2.2, − 1.6), ML7 (− 2.2, − 3.5), ML8 (− 0.8, − 3.5), MR8 (0.8, − 3.5), MR7 (2.2, − 3.5); C (0, − 4.5). Adapted from Yue et al. [46]

The experiments were performed during the reproductive season of this species. Briefly, after anesthetizing the subject using a 0.15% tricaine methanesulfonate (MS-222) solution [70, 71], 17 cortical electroencephalogram (EEG) recording electrodes, consisting of miniature stainless steel screws (φ 0.5 mm), were implanted in the skull. Sixteen electrodes were distributed in the left and right sides of telencephalon (TL1, TR1, TL2, TR2, TL3, TR3), diencephalon (DL4, DR4) and mesencephalon (ML5, MR5, ML6, MR6, ML7, MR7, ML8, MR8), respectively. The reference electrode (C) was placed on the cerebellum (Fig. 1). All electrode leads were formvar-insulated nichrome wires with one end interwined tightly around the screws and the other end tin soldered to the female-pins of an electrical connector. Electrodes were fixed to the skull with dental acrylic. The connector was covered with a self-sealing membrane (Parafilm® M; Chicago, USA) that was water-proof and located about 1 cm above the head of the animal. Finally, the skin edges and muscles surrounding the wound were treated with the ointment with triple antibiotic and pain relief (CVS pharmacy, Woonsocket, RI, USA) to prevent infection and discomfort. Each frog was housed individually for 6 days for recovery before conducting further experiments. After all experiments were completed, the subjects were euthanized by overdose of MS-222 and electrode localizations were confirmed by injecting hematoxylin dye through the skull holes in which the electrodes were installed previously [68].

### Recording conditions

An opaque plastic tank (80 × 60 cm^2^ and 60 cm deep) containing mud and water was placed in a soundproof and electromagnetically shielded chamber (background noise 24.3 ± 0.7 dB). An infrared camera with a motion detector was mounted centrally about one meter above the tank for monitoring the subjects’ movement behaviors. Electrophysiological signals were recorded with a signal acquisition system (OmniPlex 64-D, Plexon, USA). And that the sampling rate was set to 1000 Hz.

### Stimuli and paradigm

**Fig. 2:**
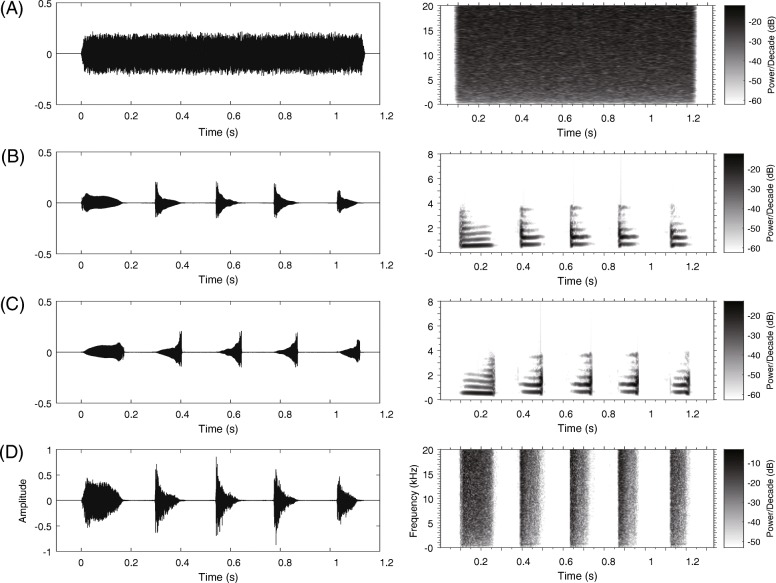
Waveforms and spectrograms of the four stimuli: **a** White noise (WN); **b** the original call (OC); **c** the version with each original note reversed (only spectral characteristics remained, SC); **d** the version with white noise enveloped by the original note (only temporal characteristics remained, TC)

Time-reversed calls have been used widely in both behavioral and neurophysiological studies because they contain the same frequencies at the same relative amplitudes as the natural calls although they show frequency modulated (FM) sweeps of reversed order for FM calls [72]. In the present study, four stimuli were used: white noise (WN), a conspecific advertisement call, its reverse version (i.e. each note of the call was reversed so that most spectral attributes of the call was preserved, SC) and its envelope version (i.e. the call envelope filled with white noise so that the most temporal attributes of the call was preserved, TC). The acoustic recording used as playback call was subject to the following criteria: (1) the call contained five notes, which is equal to the mean number of notes in natural male calls and (2) the temporal and frequency parameters of the call were close to the population average. WN without any species-specific temporal-spectral features was constructed and its duration equaled to the duration of the conspecific calls (about 1.2 s), shaped with rise and fall time sinusoidal periods of 10 ms (Fig. 2). Stimuli were played back to subjects via two portable field speakers (SME-AFS, Saul Mineroff Electronics, Elmont, NY, USA) that were placed equidistantly from the opposite ends of the experimental tank. Each stimulus was presented through the two speakers simultaneously at 65 dB SPL (re 20 μPa, C-weighting, fast response; Aihua, AWA6291; Hangzhou, China) measured at the center of the tank, approximately equals to the mean of natural sound pressure level of male calls [38]. Under these conditions, the sound level distribution at the bottom of the bank was close to a quasi-free sound field. Furthermore, subjects usually remained motionless at one corner of the tank throughout the experiments. It is highly unlikely that the tiny differences in the stimulus amplitude across the tank bottom could have a significant effect on the ERP measures.

The oddball paradigm was used in the present study with WN as the standard stimulus and others as the deviant stimuli, in which the probability of presentation for the standard stimulus was 70% and that for each deviant was 10%. Thus, for each subject a total of 1000 stimulus presentations with each deviant stimulus presented 100 times were broadcasted in a random order within three trial blocks. Randomization was constrained to prevent more than three deviant stimuli from within the same acoustic category being presented successively. A trigger pulse was sent to the signal acquisition system at every stimulus onset through the parallel port for further time-locking analysis. Because the influence of target stimulus probability on P3 amplitude would wane considerably under longer inter-stimulus intervals (ISI) in humans [73], the ISI less than 2 s was used in most animal studies [45, 64, 74]. In this study, the ISI was set to 1.5 s although the mean natural inter-call interval of the music frogs is 3.3 s [41]. Consequently, the session lasted about 50 min with 5 min breaks between blocks so that the subjects would not become fatigued [75].

### ERP signal collection and measurement

After postoperative recovery for 6 days, the subject was placed in the experimental tank and connected to the signal acquisition system for about 24 h habituation. Then the EEG signal and behavioral data were collected according to the above described auditory stimulation paradigm. In order to eliminate the effects of digestion, the subject was not fed during the experimental period. To extract ERP components, EEG recordings were filtered offline using a band-pass filter at 0.25–25 Hz and a notch filter to eliminate possible interference at 50 Hz before averaging the stimulus-locked EEG epochs. The EEG signals were divided into epochs with a duration of 700 ms, including a prestimulus baseline of 200 ms. All single EEG trials were inspected visually and trials with muscle artifacts and electrode drifts were removed from all further analysis. Accepted trials were averaged according to stimulus types and channels within each session.

For each component, the peak was found in the grand average ERP waveforms for each stimulus and each channel. Then the median was calculated regardless of stimuli and channels, and that the time window with 100 ms in width was defined with the median as the midpoint. Similar to other studies [45, 76–79], the auditory ERP component N1 was defined as the mean amplitude during latency intervals of 30–130 ms, P2 during intervals of 150–250 ms and P3a during intervals of 250–350 ms after stimulus onset. The latency was determined by the “50 percent area latency measure” for each ERP component [52], i.e. measuring the area under the curve within the time windows and finding the time point that divided this area into equal halves. Since difference waveform can be used to compare the relative variation between the ERP responses to the different deviants, they were obtained by subtracting the component amplitude in response to WN from the amplitude in response to various versions of conspecific calls. Then the amplitude and latency of each ERP component acquired from the difference waveforms (OC-WN, SC-WN and TC-WN) were subjected to further statistical analyses.

### Statistical analyses

The Shapiro-Wilk *W* test and Levene’s test were applied to estimate the normality of the distribution and the homogeneity of variances of the amplitudes and latencies of N1, P2 and P3a, respectively. Since the number of levels of an independent variable has been suggested to be less than eight [80], the amplitudes and latencies of ERP components were statistically analyzed for the telencephalon, diencephalon and mesencephalon respectively. A three-factor repeated measured ANOVA was conducted with the variables of “sex” (male/female), “stimulus” (OC/SC/TC) and “channel” (TL1, TR1, TL2, TR2, TL3 and TR3 for the telencephalon; DL4 and DR4 for the diencephalon; ML5, MR5, ML6, MR6, ML7, MR7, ML8 and MR8 for the mesencephalon). Both main effects and interactions were examined; if ANOVAs returned a significant difference, the data would be further tested for multiple comparisons using the least significant difference test. If the interaction was significant, simple effects analysis would be applied. Greenhouse-Geisser epsilon (*ε*) values would be employed when the null hypothesis of mauchly’s test of sphericity was violated. Effect size was decided by partial *η*^*2*^ (partial *η*^*2*^ = 0.20 is set as a small, 0.50 as a medium and 0.80 as a large effect size, respectively) [81]. SPSS software (release 20.0) was applied for the statistical analysis with the significance level of *p* < 0.05.

## Results

**Fig. 3:**
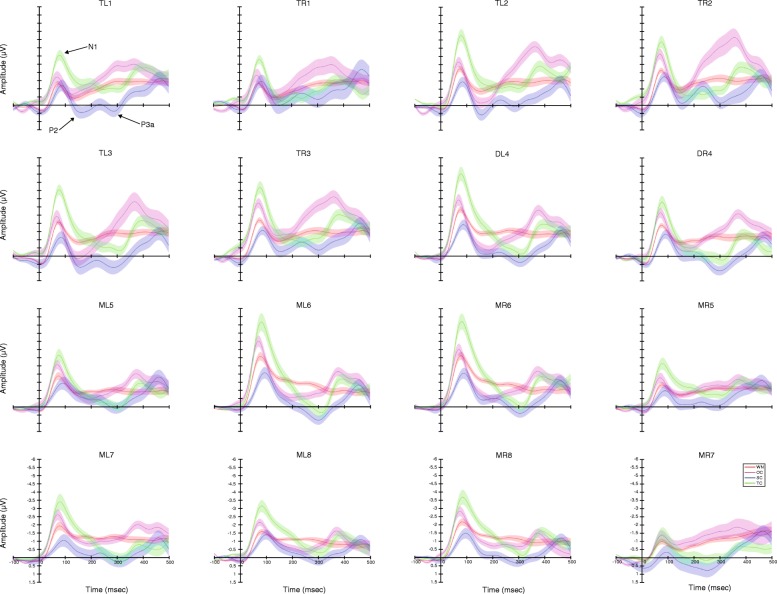
Grand average ERP waveforms with half of the standard errors for different brain regions during playbacks of white noise (WN), the original call (OC), the version with each original note reversed (only spectral characteristics remained, SC); the version with white noise enveloped by the original note (only temporal characteristics remained, TC), respectively

**Fig. 4:**
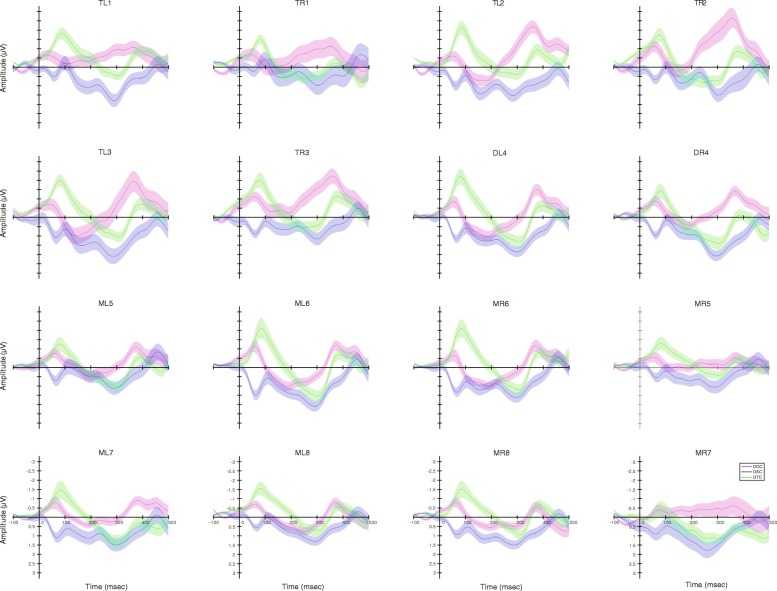
Grand average of difference waveforms with half of the standard errors for different brain regions during playbacks of the original call (DOC), the version with each original note reversed (only spectral characteristics remained, DSC); the version with white noise enveloped by the original note (only temporal characteristics remained, DTC), respectively

The grand average of the original and difference waveforms are shown in Figs. 3 and 4, respectively. There were significant differences among stimuli and sexes but not brain structures in amplitude rather than latency for each ERP component, respectively. Furthermore, SC compared with TC could elicit a more similar response to OC (Table 1).

**Table 1:** The differences between OC and SC or TC (OC-SC and OC-TC) for each ERP component

ERP component	brain region	OC-SC	OC-TC
N1	Telencephalon	−0.9606	1.0952
	Diencephalon	−1.0384	1.6570
	Mesencephalon	−1.0701	1.1728
P2	Telencephalon	−0.8183	1.0006
	Diencephalon	−0.5074	1.5998
	Mesencephalon	−0.5834	1.1094
P3a	Telencephalon	−2.5335	−1.4414
	Diencephalon	−1.9293	−0.3262
	Mesencephalon	−1.5394	−0.2660

The raw data was pooled regardless of ‘sex’ and averaged over different channels because of no significant main effect for the factors ‘sex’ and ‘channel’. Then the difference between OC and SC (OC-SC) and the difference OC and TC (OC-TC) were calculated for telencephalon, diencephalon and mesencephalon respectively

### The amplitude and latency of the N1 component

**Fig. 5:**
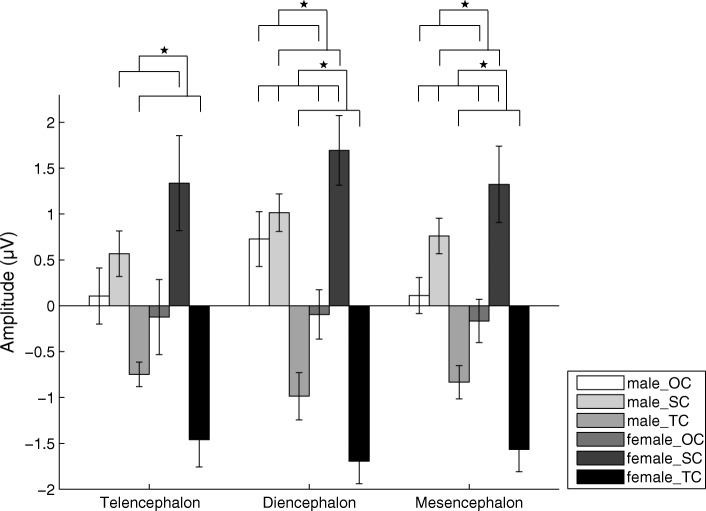
Means and standard errors for N1 amplitudes during playbacks of the three deviant stimuli for the telencephalon, diencephalon and mesencephalon respectively. OC, the original call; SC, the version with each original note reversed (only spectral characteristics remained); TC, the version with white noise enveloped by the original note (only temporal characteristics remained)

The analysis for the N1 amplitude showed that there was significant main effect for the factor “stimulus” for the telencephalon (F(2,28) = 6.046, Partial *η*^2^ = 0.302, *p* = 0.007), diencephalon (F(2,28) = 18.626, Partial *η*^2^ = 0.571, *p* < 0.001) and mesencephalon (F(2, 28) = 14.442, partial *η*^2^ = 0.508, *p* < 0.001), respectively. However, there was no significant main effect for the factors “sex” (F(1,14) = 0.007, Partial *η*^2^ = 0.000, *p* = 0.935 for the telencephalon; F(1,14) = 0.219, Partial *η*^2^ = 0.015, *p* = 0.647 for the diencephalon; and F(1,14) = 0.076, Partial *η*^2^ = 0.005, *p* = 0.787 for the mesencephalon) and “channel” (F(5,70) = 0.720, *ε* = 0.489, Partial *η*^2^ = 0.049, *p* = 0.520 for the telencephalon; F(1,14) = 1.003, Partial *η*^2^ = 0.067, *p* = 0.334 for the diencephalon; and F(7,98) = 0.851, *ε* = 0.403, Partial *η*^2^ = 0.057, *p* = 0.469 for the mesencephalon). Multiple comparisons showed that the N1 amplitudes evoked by TC were significantly greater than those evoked by OC and SC although the difference between OC and TC did not reach statistical significance for the telencephalon, while the N1 amplitudes evoked by OC was significantly higher than that by SC for the diencephalon and mesencephalon (*p* < 0.05; Fig. 5 and Table 2). In addition, for N1 latency there was no significant main effect or interaction for any factor.

**Table 2:** Results of ANOVAs for the amplitudes of N1, P2 and P3a with respect to the three factors for the telencephalon, diencephalon and mesencephalon respectively

	for the telencephalon/(2,28),(5,70),(1,14)					for the diencephalon/(2,28),(1,14),(1,14)					for the mesencephalon/(2,28),(7,98),(1,14)				
	*F*	***ε***	*p*	*η* ^2^	LSD	*F*	***ε***	*p*	*η* ^2^	LSD	*F*	***ε***	*p*	*η* ^2^	LSD
*N1*															
stimulus	6.046	NA	0.007*	0.302	TC > SC	18.626	NA	0.000**	0.571	TC > OC > SC	14.442	NA	0.000**	0.508	TC > OC > SC
channel	0.720	0.489	0.520	0.049	NA	1.003	NA	0.334	0.067	NA	0.851	0.403	0.469	0.057	NA
sex	0.007	NA	0.935	0.000	NA	0.219	NA	0.647	0.015	NA	0.076	NA	0.787	0.005	NA
interact	0.814	NA	0.453	0.055	NA	1.762	NA	0.190	0.112	NA	1.242	NA	0.304	0.081	NA
*P2*															
stimulus	5.064	NA	0.013*	0.266	SC > TC	8.003	NA	0.002*	0.364	OC,SC > TC	5.844	NA	0.008*	0.294	OC,SC > TC
channel	1.885	0.631	0.143	0.119	NA	0.314	NA	0.584	0.022	NA	0.852	0.392	0.465	0.057	NA
sex	0.013	NA	0.910	0.001	NA	0.374	NA	0.551	0.026	NA	0.128	NA	0.726	0.009	NA
interact	3.464	NA	0.045*	0.198	see main text	2.377	NA	0.111	0.145	NA	1.508	NA	0.239	0.097	NA
*P3a*															
stimulus	6.916	NA	0.004*	0.331	SC,TC > OC	5.943	NA	0.007*	0.298	SC > OC,TC	4.365	NA	0.022*	0.238	SC > OC,TC
channel	0.697	0.560	0.550	0.047	NA	1.488	NA	0.243	0.096	NA	2.054	0.422	0.122	0.128	NA
sex	0.822	NA	0.380	0.055	NA	1.178	NA	0.296	0.078	NA	0.258	NA	0.619	0.018	NA
interact	6.386	NA	0.005*	0.313	see main text	3.642	NA	0.039*	0.206	see main text	1.763	NA	0.190	0.112	NA

*Note:* The symbols ‘>’ denote that the amplitudes of ERP components evoked by the acoustic stimulus on the left side of ‘>’ are significantly larger than those on the right side, and no significant difference exists among the corresponding conditions on the same side of ‘>’ for each case. The degrees of freedom are shown after the brain regions for the three factors respectively. Note that only significant interactions are shown. ∗ *p <* 0.05, ∗∗ *p <* 0.001. Abbreviations: *F* is the *F*-value from ANOVA; *ε*, the values of epsilon of Greenhouse-Geisser correction; LSD, least-significant difference test; OC, the original note; SC, the version with each original note reversed (only spectral characteristics remained); TC, the version with white noise enveloped by the original note (only temporal characteristics remained); interact, the interaction between the factors “stimulus” and “sex”

### The amplitude and latency of the P2 component

**Fig. 6:**
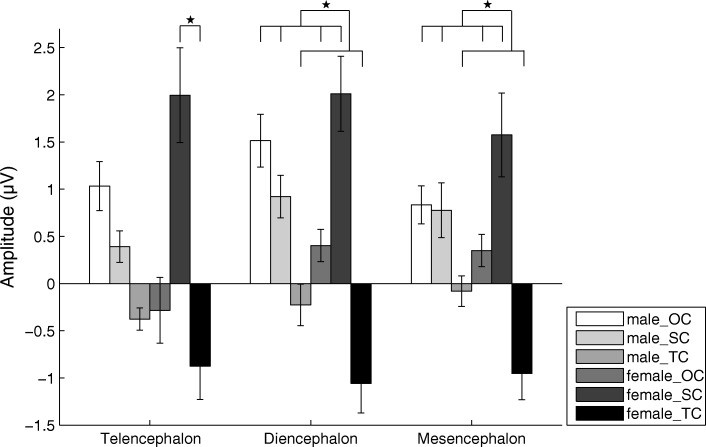
Means and standard errors for P2 amplitudes during playbacks of the three deviant stimuli for the telencephalon, diencephalon and mesencephalon respectively. OC, the original call; SC, the version with each original note reversed (only spectral characteristics remained); TC, the version with white noise enveloped by the original note (only temporal characteristics remained)

For the P2 amplitude, there was significant main effect for the factor “stimulus” for the telencephalon (F(2, 28) = 5.064, partial *η*^2^ = 0.266, *p* = 0.013), diencephalon (F(2, 28) = 8.003, partial *η*^2^ = 0.364, *p* = 0.002) and mesencephalon (F(2, 28) = 5.844, partial *η*^2^ = 0.294, *p* = 0.008), respectively. However, there was no significant main effect for the factors “sex” (F(1,14) = 0.013, Partial *η*^2^ = 0.001, *p* = 0.910 for the telencephalon; F(1,14) = 0.374, Partial *η*^2^ = 0.026, *p* = 0.551 for the diencephalon; and F(1,14) = 0.128, Partial *η*^2^ = 0.009, *p* = 0.726 for the mesencephalon) and “channel” (F(5,70) = 1.885, *ε* = 0.631, Partial *η*^2^ = 0.119, *p* = 0.143 for the telencephalon; F(1,14) = 0.314, Partial *η*^2^ = 0.022, *p* = 0.584 for the diencephalon; and F(7,98) = 0.852, *ε* = 0.392, Partial *η*^2^ = 0.057, *p* = 0.465 for the mesencephalon). And that the interaction between “sex” and “stimulus” was significant (F(2, 28) = 3.464, partial *η*^2^ = 0.198, *p* = 0.045) for the telencephalon. Simple effects analysis showed that the P2 amplitude evoked by SC was significantly higher than that by TC in females (*p* < 0.05; Fig. 6 and Table 2). For the diencephalon and mesencephalon, the P2 amplitudes evoked by OC and SC were significantly higher than that evoked by TC (*p* < 0.05; Fig. 6 and Table 2). Similarly, for P2 latency there was no significant main effect or interaction for any factor.

### The amplitude and latency of the P3a component

**Fig. 7:**
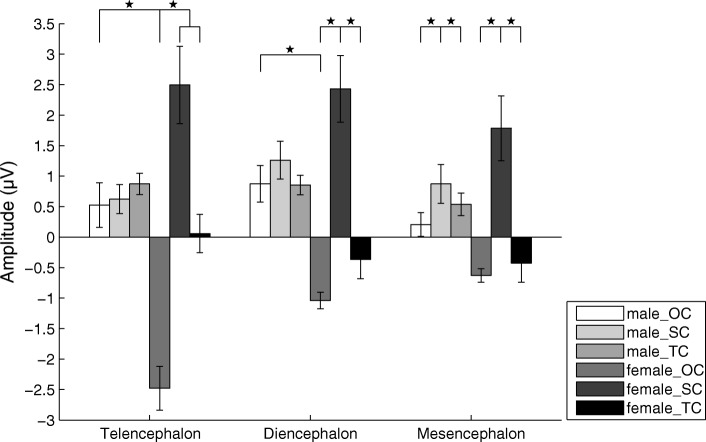
Means and standard errors for P3a amplitudes during playbacks of the three deviant stimuli for the telencephalon, diencephalon and mesencephalon respectively. OC, the original call; SC, the version with each original note reversed (only spectral characteristics remained); TC, the version with white noise enveloped by the original note (only temporal characteristics remained)

For the P3a amplitude in the telencephalon, there was significant main effect for the factor “stimulus” (F(2, 28) = 6.916, partial *η*^2^ = 0.331, *p* = 0.004) but not the factors “sex” (F(1, 14) = 0.822, partial *η*^2^ = 0.055, *p* = 0.380) and “channel” (F(5, 70) = 0.697, *ε* = 0.560, partial *η*^2^ = 0.047, *p* = 0.550). Moreover, the interaction between “sex” and “stimulus” was significant (F(2, 28) = 6.386, partial *η*^2^ = 0.313, *p* = 0.005). The P3a amplitudes evoked by SC and TC were significantly higher than that evoked by OC in females (*p* < 0.05; Fig. 7 and Table 2), and that the P3a amplitude in males evoked by OC was significantly higher than that evoked in females. For the diencephalon, there was significant main effect for the factor “stimulus” (F(2, 28) = 5.943, partial *η*^2^ = 0.298, *p* = 0.007) but not the factors “sex” (F(1, 14) = 1.178, partial *η*^2^ = 0.078, *p* = 0.296) and “channel” (F(1, 14) = 1.488, partial *η*^2^ = 0.096, *p* = 0.243). Moreover, the interaction between “sex” and “stimulus” was significant (F(2, 28) = 3.642, partial *η*^2^ = 0.206, *p* = 0.039). The P3a amplitude evoked by SC was significantly higher than those evoked by OC and TC in females (*p* < 0.05; Fig. 7 and Table 2), and that the P3a amplitude in males evoked by OC was significantly higher than that evoked in females. For the mesencephalon, there was significant main effect for the factor “stimulus” (F(2, 28) = 4.365, partial *η*^2^ = 0.238, *p* = 0.022) but not the factors “sex” (F(1, 14) = 0.258, partial *η*^2^ = 0.018, *p* = 0.619) and “channel” (F(7, 98) = 2.054, *ε* = 0.422, partial *η*^2^ = 0.128, *p* = 0.122). The P3a amplitude evoked by SC was significantly higher than those evoked by OC and TC (*p* < 0.05; Fig. 7 and Table 2). Similarly, for P3a latency there was no significant main effect or interaction for any factor.

## Discussion

The present study showed that when the three deviant stimuli consisting of OC, SC and TC were presented 1) although some differences did not reach statistical significance for the telencephalon, the N1 amplitude evoked by TC was significantly greater than those evoked by OC and SC, while the N1 amplitude evoked by OC was significant greater than that by SC; 2) the P2 amplitudes evoked by OC and SC were significantly greater than that by TC although the difference between OC and TC did not reach statistical significance for the telencephalon; 3) the P3a amplitudes evoked by SC and TC were significantly higher than by OC although the differences between TC and OC did not reach statistical significance for the diencephalon and mesencephalon; in addition, P3a amplitudes in the forebrain evoked by OC were significantly higher in males than in females. These results are consistent with the hypothesis that auditory processing of conspecific vocalization prefers to spectral features compared with temporal ones in the music frog. Moreover, the current results suggest that the neural processing for auditory perception is sexually dimorphic.

### Neural processing of conspecific vocalization prefers to spectral features

Spectral and temporal processing refers to the transformations in how the spectral and temporal structures of sounds is represented in the central auditory system. In the present study, significant differences in N1 and P2 amplitudes were found exclusively between TC and other two stimuli in most conditions, although N1 amplitudes evoked by OC were also significantly higher than those by SC. In addition, the absolute values of difference of N1 or P2 amplitudes between OC and SC were smaller than those between OC and TC (Table 1), thus compared with TC the neural responses to SC were more similar to those for OC. Although SC shows reversed order of FM sweeps compared with OC, SC contains the same frequencies at the same relative amplitudes as OC. Accordingly, the present results were consistent with the prediction that more similar ERP components would be evoked by OC and SC if neural processing of conspecific vocalization depends on spectral features primarily. Compared with other deviant stimuli, higher N1 amplitude evoked by TC is consistent with the idea that the negative N1 waves can be affected by selective attention which enhances the perception of high-priority stimuli at the expense of other stimuli in the environment [53, 82]. Animals usually pay attention to conspecific sounds with high salience and generally maintain alertness to absolute novelty of sounds (according to past auditory experience of the subject) which may be associated with danger [83–85], and that the stimuli with high emotional valence may capture attention [86, 87]. Accordingly, this strong selective pressure would likely result in a large “N1 effect of selective attention” [88]. Since more similar N1 was evoked by OC and SC, higher N1 amplitude evoked by TC would be more likely resulted from absolute novelty rather than conspecific salience involved in this sound. In addition, N1 is known to be sensitive to onset parameters [76] such as rise time with N1 peak amplitude reducing when stimulus rise time increases [89]. Consistent with this, the present results showed that the N1 amplitude evoked by SC with longest rise time was smallest.

The P2 component reflects the process of signal evaluation and classification, and is thought to be a connected with the memory processing and will compare the real-time perception input with the memory [54, 90, 91]. Moreover, its amplitude enhancement can result from prolonged training in mammals. Therefore P2 amplitude can be enhanced by familiarity or similarity between the target and current stimulus [54, 90–93], i.e. more familiar stimuli will evoke larger P2 waveforms [94]. Since humanlike auditory ERP components may indicate similar brain functions because of important conserved neuroanatomical features in vertebrate brain [66, 67], the present results showing OC and SC evoked higher P2 amplitude than TC did suggest SC compared with TC seemed to be more like conspecific vocalization. However, future research is required to verify it via behavioral experiments. In addition, the acoustic complexity can effect on the P2 amplitude significantly [95]. If this is the case, TC would be expected to evoke a relatively larger P2 amplitude because of its most complexity. However, OC and SC actually evoked a larger P2 amplitudes compared with TC, so it is likely that these results for P2 did not occur because of the presumed effects of complexity, thus implying that the similar spectral characteristics of sounds are the key factors for P2 profiles in the music frogs. Thus, neural processing of conspecific vocalization may prefer to spectral features in this species. This speculation has been verified partly by discriminant function analysis of calls in the music frog [38], which show the spectral features may provide more sufficient information for individual recognition compared with the temporal ones.

At the individual level, some kinds of acoustic properties of advertisement calls typically show very little variation (static properties) and others are highly variable (dynamic properties) [96]. Variability in static properties is usually constrained within individual, therefore these properties are highly invariant from call to call within and between bouts of calling by an individual. Typically these properties include spectral features such as the fundamental frequency or dominant frequency or carrier frequency and fine-scale temporal properties such as the duration, rise-fall features and repetition rate of the short sounds (pulses) [96]. In contrast, anuran individuals readily alter gross-temporal properties of advertisement calls within and between calling bouts, such as the rate of calling, duration of calls or call-notes and rate of call-note production [97]. Since such signals may be more easily detected against the chorus background, females usually prefer calls with longer duration and higher rate. However, for an individual of the music frogs the spectral attributes of advertisement call remain relatively stable compared with the temporal ones [38, 41, 98], suggesting the static properties in this species include spectral features primarily rather than temporal characteristics. Taken together, static variables, i.e. spectral features in the music frogs, are presumably more important for species discrimination and individual recognition, although dynamic variables like call rate and call duration are indicative of motivation or quality of the emitter [97] and may play an important role in female choice.

### Auditory perception on temporal and spectral features of calls exhibits sexual dimorphism

Sexually dimorphic behaviors are widespread in vocal animals such as insects, birds and anurans [48, 65, 99–105]. In general, females may be mute or exhibit a severely limited vocal repertoire while males are typically highly vocal and generally produce complex species-specific vocalizations to attract females for breeding, as well as to deter rivals [24, 106]. Moreover, males and females often react differently in response to conspecific calls, during which males are much more likely than females to respond to signals which vary from the species’ norm [101]. These behavioral differences depend on neural systems that are sex-specific or common to males and females but potentially regulate a number of behaviors differently [107]. In other words, sex differences in auditory processing may reflect differences in the requirement for processing sex-specific aspects of vocal signals [97].

The present results show that the P3a amplitudes evoked by OC are significantly greater for males than females regardless of brain area, although the differences for the mesencephalon did not reach statistical significance (Fig. 7). P3a is usually evoked by the novel stimulus (relative novelty) with small proportion of occurrence [108]. Its amplitude is appears to be a reflection of automatic detection of a different stimulus or stimulus relative novelty, i.e. novel or more salient differences between standard and deviant stimuli produce larger P3a waves [60]. Furthermore, familiar sounds evoke smaller P3a compared with unfamiliar ones [61]. In this way, SC would be expected to evoke a relatively larger P3a amplitude because of sound familiarity for OC and almost identical spectral attributes between standard and TC.

Previous study showed that males are more permissive than females in their responses to signals [101]. Consistent with this idea, egr-1 expression in the auditory midbrain of male túngara frogs (*Physalaemus pustulosus*) increases in response to either conspecific or heterospecific calls but only increases in response to conspecific signals in females [103]. Similarly, a previous study of the auditory midbrain in large odorous frogs (*Odorrana graminea*) showed that the most sensitive frequency range in males is almost double bandwidth of females [109]. These results imply that in at least some species males may process more acoustic information than females when they are under the same auditory scene. Thus, more relatively novel or more salient differences between standard and deviant stimuli may be detected in males compared with females during acoustic signal perception. These sex differences are consistent with the fact that the cost of not responding to a potential sexual signal would be greater in males than females while the cost of responding inappropriately to sexual solicitation signals would be greater in females than males [110, 111]. Interestingly, the auditory brainstem response amplitude of male house sparrows (*Passer domesticus*), increases at a greater rate than that of females as the amplitude of the stimulus increases [16]. These findings, including the present results, suggest that sex differences in auditory processing occur but that the exact nature of these differences is both species specific and time specific, and that sexual dimorphism in auditory perception evolved in diverse vocal species.

The present results also show that the P3a amplitudes evoked by SC and TC in the telencephalon and diencephalon are greater than that by OC in females but not males. These results are generally consistent with other studies on P3a, showing less relative novelty or more familiarity in sounds elicit decreased P3a amplitude while more relative novelty or less familiarity in sounds elicit increased P3a amplitude [61] and with the idea that the forebrain may play an important role in auditory perception [65]. No specific sensory areas in the anuran telencephalon appear homologous to the auditory areas of the amniote telencephalon insofar as the anuran pallium is not parcellated into discrete functional areas, although widespread connections linking forebrain neurons to motor and/or endocrine systems and limbic structures exist [112]. Thus the sex differences in P3a amplitude in the telencephalon observed in the present study may reflect the differential effects in males and females of selection pressures associated with identifying male conspecific call differences and in decision making associated with responding to male calls. Consistent with this, simple stimuli such as clicks generally fail to excite cells in the frog telencephalon [113]; in contrast, complex signals similar to natural calls can induce large neuronal responses in the striatum and medial pallium. Lesions of the striatum, superficial and deep thalamic structures may disrupt vocal recognition [114], indicating that telencephalic and thalamic areas play important roles in call recognition. Consequently, more telencephalic resources appear to be involved in higher level cognition functions such as mate choice in females than in males during the breeding season.

## Conclusion

Taken together, we found evidence that more similar ERP components were evoked by the original call and its transformation version with most spectral features preserved, compared with the other version with temporal characteristics preserved. Moreover, the P3a amplitudes in the forebrain evoked by the original call were significantly higher in males than in females. These results suggest neural processing for conspecific vocalization may prefer to the spectral features of species-specific call in the music frogs, prompting speculation that the spectral features may play more important roles in auditory object perception or vocal communication in this species. In addition, the neural processing for auditory perception is sexually dimorphic.
